# 9-Ethyl-2,3-dihydro-9*H*-carbazol-4(1*H*)-one

**DOI:** 10.1107/S1600536808024318

**Published:** 2008-08-06

**Authors:** S. Murugavel, G. Ganesh, A. Subbiah Pandi, Ramalingam Murugan, S. Sriman Narayanan

**Affiliations:** a Department of Physics, Thanthai Periyar Government Institute of Technology, Vellore 632 002, India; b Department of Physics, SMK Fomra Institute of Technology, Thaiyur, Chennai 603 103, India; c Department of Physics, Presidency College (Autonomous), Chennai 600 005, India; d Department of Analytical Chemistry, University of Madras, Guindy Campus, Chennai 600 025, India

## Abstract

In the title compound, C_28_H_30_N_2_O_2_, the cyclo­hexene ring system adopts a sofa conformation. The crystal structure is stabilized by C—H⋯O inter­actions between methyl H atoms of the ethyl substituents and the O atoms of carbonyl groups of adjacent mol­ecules, and by an inter­molecular carbon­yl–carbonyl inter­actions [3.207 (2) Å]

## Related literature

For related literature, see: Abraham (1975 ▶); Govindasamy *et al.* (1999 ▶); Hewlins *et al.* (1984 ▶); Kansal *et al.* (1986 ▶); Mi *et al.* (2003 ▶); Nardelli (1983 ▶); Phillipson & Zenk (1980 ▶); Saxton (1983 ▶); Allen *et al.* (1998 ▶); Cremer & Pople (1975 ▶); Mohanakrishnan & Srinivasasan (1995 ▶ ▶, 1995 ▶ ▶).
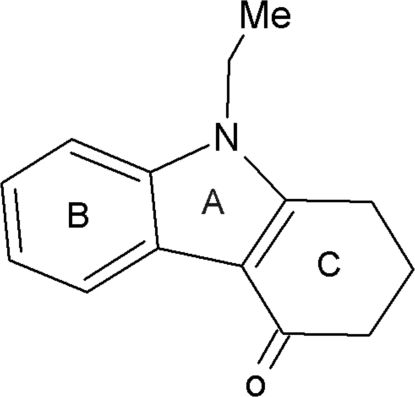


## Experimental

### 

#### Crystal data

C_14_H_15_NO
*M* *_r_* = 213.27
Monoclinic, 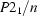

*a* = 8.3742 (6) Å
*b* = 17.033 (1) Å
*c* = 8.6083 (5) Å
β = 116.432 (3)°
*V* = 1099.51 (12) Å^3^
*Z* = 4
Mo *K*α radiation
μ = 0.08 mm^−1^
*T* = 293 (2) K
0.21 × 0.19 × 0.17 mm

#### Data collection

Bruker APEXII CCD area-detector diffractometer
Absorption correction: none
11070 measured reflections
2334 independent reflections
1898 reflections with *I* > 2σ(*I*)
*R*_int_ = 0.029

#### Refinement

*R*[*F* ^2^ > 2σ(*F* ^2^)] = 0.040
*wR*(*F* ^2^) = 0.105
*S* = 1.03
2334 reflections
146 parameters
H-atom parameters constrained
Δρ_max_ = 0.15 e Å^−3^
Δρ_min_ = −0.20 e Å^−3^

### 

Data collection: *APEX2* (Bruker, 2004 ▶); cell refinement: *APEX2*; data reduction: *SAINT* (Bruker, 2004 ▶); program(s) used to solve structure: *SHELXS97* (Sheldrick, 2008 ▶); program(s) used to refine structure: *SHELXL97* (Sheldrick, 2008 ▶); molecular graphics: *ORTEP-3* (Farrugia, 1997 ▶); software used to prepare material for publication: *SHELXL97* and *PLATON* (Spek, 2003 ▶).

## Supplementary Material

Crystal structure: contains datablocks global, I. DOI: 10.1107/S1600536808024318/lx2064sup1.cif

Structure factors: contains datablocks I. DOI: 10.1107/S1600536808024318/lx2064Isup2.hkl

Additional supplementary materials:  crystallographic information; 3D view; checkCIF report

## Figures and Tables

**Table 1:** Hydrogen-bond geometry (Å, °)

*D*—H⋯*A*	*D*—H	H⋯*A*	*D*⋯*A*	*D*—H⋯*A*
C14—H14*A*⋯O1^i^	0.96	2.60	3.549 (2)	170

Symmetry code: (i) 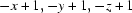.

**Fig. 1.**
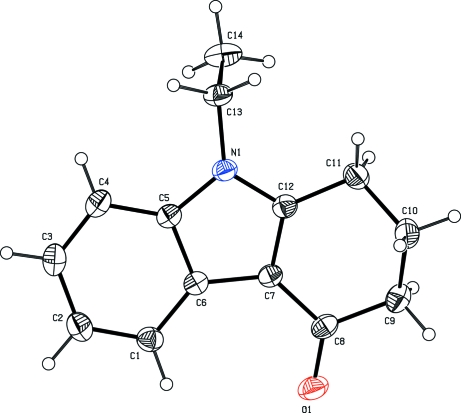
The molecular structure of title compound showing 30% probability displacement ellipsoids.

**Fig. 2.**
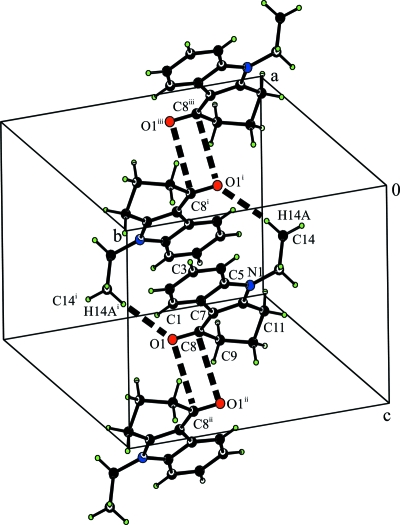
C—H···O and C···O interaction(dotted lines) in the title compound. [Symmetry code: (i) -x+1, -y+1, -z+1; (ii) -x+1, -y+1, -z+2; (iii) x, y, z-1.]

## supplementary crystallographic information

### Comment

Carbazole derivatives exhibit good charge transfer and hole transporting properties, which are being explored for a multitude of optoelectronic and photocatalytic applications, including organic light emitting diodes (OLEDs) (Mi *et al.*,  2003). In carbazole derivatives, the preliminary study shows that the presence of oxygenated substituents increases their biological activity (Hewlins, Oliveira-Campos & Shannon, 1984).  The 2,3-disubstituted indoles have been used as bidentate synthons for the synthesis of various medicinally important carbazole alkaloids (Mohanakrishnan & Srinivasan, 1995). Intercalation between the base pairs in DNA has been implicated for their anticancer activity. It was conceived that the benzo[b] carbazoles as isosteric analogs of pyrido[4,3-b]carbazoles, with oxygenated D-ring could mimic the anti-cancer activity of ellipticine. So it was of interest to study the anticancer activity of D-ring oxygenated benzo[b]carbazoles as it is believed that these molecules could form a stable intercalation complex with DNA (Kansal & Potier, 1986). Tetrahydrocarbazole derivatives are present in the framework of indole-type alkaloids of biological interest (Phillipson & Zenk, 1980; Saxton, 1983; Abraham, 1975). Here we report the crystal and molecular structure of the title compound, 9-ethyl-1,2,3-trihydrocarbazol-4(2H)-one (Fig. 1).

The planarities of  rings A and C are fairly good. The bond lengths C8—O1, N1—C5 and N1—C12 are normal and comparable with the corresponding values observed in the related structure. (Govindasamy *et al.*,  1999). The atom O1 deviates by -0.033 (1) Å from the least-squares plane of the ring C. The cyclohexane ring of the carbazole moiety adopts sofa  conformation, with lowest displacement asymmetric parameter (Nardelli, 1983), ΔC_s_(C7) = 2.26 (1) °, and puckering parameter (Cremer & Popple, 1975) q2 = 0.373 (2) Å and φ = 359.1 (2) °. The crystal packing (Fig. 2) is stabilized by a C—H···O interaction between a methyl H atom of the ethyl substituent and the oxygen of the carbonyl group of an adjacent molecule, with a C14—H14A···O1^i^ separation of 2.60 Å (Fig. 2 and Table 1; symmetry code as in Fig. 2). The molecular packing (Fig. 2) is further stabilized by a type-II carbonyl-carbonyl interaction (Allen *et al.*,  1998), with C8···O1^ii^ and O1···C8^ii^ distance of 3.207 (2) Å (symmetry code as in Fig. 2).

### Experimental

A mixture of (0.5 g, 1.0 mol), ethyl bromide (0.18 g, 1.0 mol) and potassium carbonate (2.0 g) in 1,4-dioxane (10 ml) was refluxed for ca. 5.0 h. Then the reaction mixture was poured in water and then the crude solid was filtered. Single crystals suitable for X-ray diffraction were obtained by slow evaporation of a solution of the title compound in ethanol at room temperature.

### Refinement

All H atoms were fixed geometrically and allowed to ride on their parent C atoms, with C—H distances fixed in the range 0.93–0.98 Å with *U*_iso_(H) = 1.5*U*_eq_(C) for methyl H 1.2*U*_eq_(C) for other H atoms.

### Crystal data

**Table:**

C_14_H_15_NO	*F*_000_ = 456
*M**_r_* = 213.27	*D*_x_ = 1.288 Mg m^−^^3^
Monoclinic, *P*2_1_/*n*	Mo *K*α radiation λ = 0.71073 Å
Hall symbol: -P 2yn	Cell parameters from 2850 reflections
*a* = 8.3742 (6) Å	θ = 20.0–26.8º
*b* = 17.033 (1) Å	µ = 0.08 mm^−^^1^
*c* = 8.6083 (5) Å	*T* = 293 (2) K
β = 116.432 (3)º	Block, colourless
*V* = 1099.51 (12) Å^3^	0.21 × 0.19 × 0.17 mm
*Z* = 4	

### Data collection

**Table:**

Bruker APEXII CCD area-detector diffractometer	2334 independent reflections
Radiation source: fine-focus sealed tube	1898 reflections with *I* > 2σ(*I*)
Monochromator: graphite	*R*_int_ = 0.029
Detector resolution: 10 pixels mm^-1^	θ_max_ = 26.8º
*T* = 293(2) K	θ_min_ = 2.4º
ω scans	*h* = −10→10
Absorption correction: none	*k* = −21→21
11070 measured reflections	*l* = −10→10

### Refinement

**Table:**

Refinement on *F*^2^	Secondary atom site location: difference Fourier map
Least-squares matrix: full	Hydrogen site location: inferred from neighbouring sites
*R*[*F*^2^ > 2σ(*F*^2^)] = 0.040	H-atom parameters constrained
*wR*(*F*^2^) = 0.105	*w* = 1/[σ^2^(*F*_o_^2^) + (0.0497*P*)^2^ + 0.2126*P*] where *P* = (*F*_o_^2^ + 2*F*_c_^2^)/3
*S* = 1.03	(Δ/σ)_max_ < 0.001
2334 reflections	Δρ_max_ = 0.15 e Å^−^^3^
146 parameters	Δρ_min_ = −0.20 e Å^−^^3^
Primary atom site location: structure-invariant direct methods	Extinction correction: none

### Special details

**Table:**

Geometry. All esds (except the esd in the dihedral angle between two l.s. planes) are estimated using the full covariance matrix. The cell esds are taken into account individually in the estimation of esds in distances, angles and torsion angles; correlations between esds in cell parameters are only used when they are defined by crystal symmetry. An approximate (isotropic) treatment of cell esds is used for estimating esds involving l.s. planes.
Refinement. Refinement of F^2^ against ALL reflections. The weighted R-factor wR and goodness of fit S are based on F^2^, conventional R-factors R are based on F, with F set to zero for negative F^2^. The threshold expression of F^2^ > 2sigma(F^2^) is used only for calculating R-factors(gt) etc. and is not relevant to the choice of reflections for refinement. R-factors based on F^2^ are statistically about twice as large as those based on F, and R- factors based on ALL data will be even larger.

### Fractional atomic coordinates and isotropic or equivalent isotropic displacement parameters (Å^2^)

**Table:**

	*x*	*y*	*z*	*U*_iso_*/*U*_eq_	
C1	0.41850 (18)	0.62548 (8)	0.62150 (18)	0.0419 (3)	
H1	0.5200	0.6388	0.7217	0.050*	
C2	0.3279 (2)	0.68137 (8)	0.4981 (2)	0.0506 (4)	
H2	0.3685	0.7330	0.5160	0.061*	
C3	0.1766 (2)	0.66211 (9)	0.3472 (2)	0.0527 (4)	
H3	0.1186	0.7011	0.2659	0.063*	
C4	0.11103 (19)	0.58687 (8)	0.31534 (17)	0.0457 (3)	
H4	0.0102	0.5741	0.2140	0.055*	
C5	0.20092 (16)	0.53077 (8)	0.44009 (15)	0.0359 (3)	
C6	0.35512 (16)	0.54847 (7)	0.59327 (15)	0.0343 (3)	
C7	0.40707 (16)	0.47683 (7)	0.69054 (15)	0.0350 (3)	
C8	0.55752 (16)	0.45924 (8)	0.85455 (16)	0.0392 (3)	
C9	0.56815 (19)	0.37540 (9)	0.91598 (19)	0.0490 (4)	
H9A	0.6218	0.3752	1.0417	0.059*	
H9B	0.6462	0.3461	0.8811	0.059*	
C10	0.38967 (19)	0.33358 (9)	0.84713 (19)	0.0509 (4)	
H10A	0.3178	0.3574	0.8970	0.061*	
H10B	0.4091	0.2790	0.8832	0.061*	
C11	0.28888 (19)	0.33744 (8)	0.65050 (19)	0.0465 (3)	
H11A	0.3476	0.3049	0.5989	0.056*	
H11B	0.1682	0.3182	0.6123	0.056*	
C12	0.28502 (16)	0.42042 (7)	0.59547 (16)	0.0364 (3)	
C13	0.01251 (17)	0.41064 (8)	0.30768 (17)	0.0431 (3)	
H13A	−0.0791	0.4483	0.2400	0.052*	
H13B	−0.0388	0.3740	0.3593	0.052*	
C14	0.0697 (2)	0.36655 (11)	0.1896 (2)	0.0595 (4)	
H14A	0.1250	0.4022	0.1418	0.089*	
H14B	−0.0326	0.3429	0.0973	0.089*	
H14C	0.1534	0.3264	0.2543	0.089*	
N1	0.16080 (13)	0.45195 (6)	0.44527 (13)	0.0372 (3)	
O1	0.67261 (13)	0.50731 (6)	0.93817 (12)	0.0530 (3)	

### Atomic displacement parameters (Å^2^)

**Table:**

	*U*^11^	*U*^22^	*U*^33^	*U*^12^	*U*^13^	*U*^23^
C1	0.0411 (7)	0.0438 (7)	0.0420 (7)	−0.0052 (6)	0.0196 (6)	−0.0026 (6)
C2	0.0601 (9)	0.0400 (7)	0.0568 (9)	−0.0026 (7)	0.0307 (8)	0.0030 (6)
C3	0.0602 (9)	0.0474 (8)	0.0506 (8)	0.0113 (7)	0.0248 (7)	0.0144 (7)
C4	0.0426 (8)	0.0533 (8)	0.0364 (7)	0.0082 (6)	0.0134 (6)	0.0042 (6)
C5	0.0341 (6)	0.0409 (7)	0.0335 (6)	0.0027 (5)	0.0158 (5)	−0.0019 (5)
C6	0.0321 (6)	0.0397 (7)	0.0331 (6)	0.0006 (5)	0.0164 (5)	−0.0013 (5)
C7	0.0314 (6)	0.0387 (7)	0.0336 (6)	0.0000 (5)	0.0133 (5)	−0.0012 (5)
C8	0.0312 (6)	0.0514 (8)	0.0344 (6)	0.0007 (6)	0.0140 (5)	−0.0014 (6)
C9	0.0422 (8)	0.0549 (9)	0.0430 (7)	0.0080 (6)	0.0128 (6)	0.0090 (6)
C10	0.0509 (9)	0.0447 (8)	0.0546 (8)	0.0030 (6)	0.0213 (7)	0.0113 (6)
C11	0.0443 (8)	0.0370 (7)	0.0535 (8)	−0.0003 (6)	0.0176 (6)	−0.0008 (6)
C12	0.0327 (6)	0.0393 (7)	0.0362 (6)	0.0019 (5)	0.0145 (5)	−0.0018 (5)
C13	0.0311 (7)	0.0491 (8)	0.0415 (7)	−0.0042 (6)	0.0093 (5)	−0.0086 (6)
C14	0.0462 (9)	0.0782 (11)	0.0497 (8)	−0.0101 (8)	0.0172 (7)	−0.0251 (8)
N1	0.0318 (5)	0.0389 (6)	0.0346 (6)	−0.0001 (4)	0.0092 (4)	−0.0041 (4)
O1	0.0399 (5)	0.0643 (7)	0.0420 (5)	−0.0092 (5)	0.0067 (4)	−0.0038 (5)

### Geometric parameters (Å, °)

**Table:**

C1—C2	1.376 (2)	C9—H9A	0.9700
C1—C6	1.395 (2)	C9—H9B	0.9700
C1—H1	0.9300	C10—C11	1.520 (2)
C2—C3	1.391 (2)	C10—H10A	0.9700
C2—H2	0.9300	C10—H10B	0.9700
C3—C4	1.373 (2)	C11—C12	1.486 (2)
C3—H3	0.9300	C11—H11A	0.9700
C4—C5	1.383 (2)	C11—H11B	0.9700
C4—H4	0.9300	C12—N1	1.358 (2)
C5—N1	1.390 (2)	C13—N1	1.460 (2)
C5—C6	1.408 (2)	C13—C14	1.503 (2)
C6—C7	1.434 (2)	C13—H13A	0.9700
C7—C12	1.376 (2)	C13—H13B	0.9700
C7—C8	1.445 (2)	C14—H14A	0.9600
C8—O1	1.225 (2)	C14—H14B	0.9600
C8—C9	1.512 (2)	C14—H14C	0.9600
C9—C10	1.518 (2)		
			
C2—C1—C6	118.67 (13)	C9—C10—C11	112.13 (12)
C2—C1—H1	120.7	C9—C10—H10A	109.2
C6—C1—H1	120.7	C11—C10—H10A	109.2
C1—C2—C3	121.19 (14)	C9—C10—H10B	109.2
C1—C2—H2	119.4	C11—C10—H10B	109.2
C3—C2—H2	119.4	H10A—C10—H10B	107.9
C4—C3—C2	121.57 (13)	C12—C11—C10	108.58 (11)
C4—C3—H3	119.2	C12—C11—H11A	110.0
C2—C3—H3	119.2	C10—C11—H11A	110.0
C3—C4—C5	117.31 (13)	C12—C11—H11B	110.0
C3—C4—H4	121.3	C10—C11—H11B	110.0
C5—C4—H4	121.3	H11A—C11—H11B	108.4
C4—C5—N1	129.54 (12)	N1—C12—C7	109.97 (11)
C4—C5—C6	122.31 (12)	N1—C12—C11	125.32 (11)
N1—C5—C6	108.12 (10)	C7—C12—C11	124.71 (11)
C1—C6—C5	118.94 (12)	N1—C13—C14	112.16 (11)
C1—C6—C7	134.87 (12)	N1—C13—H13A	109.2
C5—C6—C7	106.15 (11)	C14—C13—H13A	109.2
C12—C7—C6	107.16 (11)	N1—C13—H13B	109.2
C12—C7—C8	122.03 (12)	C14—C13—H13B	109.2
C6—C7—C8	130.79 (12)	H13A—C13—H13B	107.9
O1—C8—C7	123.48 (13)	C13—C14—H14A	109.5
O1—C8—C9	121.17 (12)	C13—C14—H14B	109.5
C7—C8—C9	115.32 (11)	H14A—C14—H14B	109.5
C8—C9—C10	114.35 (11)	C13—C14—H14C	109.5
C8—C9—H9A	108.7	H14A—C14—H14C	109.5
C10—C9—H9A	108.7	H14B—C14—H14C	109.5
C8—C9—H9B	108.7	C12—N1—C5	108.60 (10)
C10—C9—H9B	108.7	C12—N1—C13	126.60 (11)
H9A—C9—H9B	107.6	C5—N1—C13	124.77 (11)
			
C6—C1—C2—C3	−0.4 (2)	C7—C8—C9—C10	−26.10 (17)
C1—C2—C3—C4	0.3 (2)	C8—C9—C10—C11	53.43 (17)
C2—C3—C4—C5	0.3 (2)	C9—C10—C11—C12	−50.05 (16)
C3—C4—C5—N1	177.12 (13)	C6—C7—C12—N1	0.19 (14)
C3—C4—C5—C6	−0.9 (2)	C8—C7—C12—N1	−178.24 (11)
C2—C1—C6—C5	−0.11 (18)	C6—C7—C12—C11	−179.46 (12)
C2—C1—C6—C7	−177.39 (13)	C8—C7—C12—C11	2.1 (2)
C4—C5—C6—C1	0.80 (18)	C10—C11—C12—N1	−155.43 (12)
N1—C5—C6—C1	−177.59 (11)	C10—C11—C12—C7	24.16 (19)
C4—C5—C6—C7	178.79 (12)	C7—C12—N1—C5	0.06 (14)
N1—C5—C6—C7	0.40 (13)	C11—C12—N1—C5	179.71 (12)
C1—C6—C7—C12	177.16 (14)	C7—C12—N1—C13	178.10 (11)
C5—C6—C7—C12	−0.36 (13)	C11—C12—N1—C13	−2.2 (2)
C1—C6—C7—C8	−4.6 (2)	C4—C5—N1—C12	−178.53 (13)
C5—C6—C7—C8	177.88 (12)	C6—C5—N1—C12	−0.29 (13)
C12—C7—C8—O1	176.58 (12)	C4—C5—N1—C13	3.4 (2)
C6—C7—C8—O1	−1.4 (2)	C6—C5—N1—C13	−178.38 (11)
C12—C7—C8—C9	−1.59 (18)	C14—C13—N1—C12	−80.84 (17)
C6—C7—C8—C9	−179.60 (13)	C14—C13—N1—C5	96.90 (16)
O1—C8—C9—C10	155.68 (13)		

### Hydrogen-bond geometry (Å, °)

**Table:**

*D*—H···*A*	*D*—H	H···*A*	*D*···*A*	*D*—H···*A*
C14—H14A···O1^i^	0.96	2.60	3.549 (2)	170

Symmetry codes: (i) −*x*+1, −*y*+1, −*z*+1.
